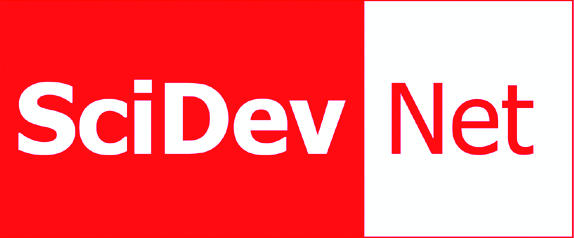# Science and Technology Communication for Development

**DOI:** 10.1371/journal.pbio.0020011

**Published:** 2004-01-20

**Authors:** David Dickson

## Abstract

The Science and Development Network (SciDev.Net) is a source of online news and analysis about the role of science and technology in meeting the needs of the developing world

Developing countries today face a wide range of needs, from more secure food supplies to cheap and effective medicines. One factor that almost all these needs have in common is that satisfying them adequately will not occur without the use of modern science. These same countries also face a range of political dilemmas—such as whether to accept the growing of genetically modified crops or how to adapt to the impact of climate change—that also require a knowledge of science, albeit of a slightly different nature. From both points of view, development can be characterised as the process of putting scientific and technical knowledge into practice. Conversely, it is important that building the capacity to absorb and make use of scientific and technical knowledge must be placed at the heart of the development aid efforts if these are to be successful in achieving their goals.

But knowledge will not reach those who can benefit from it unless it has been effectively communicated to individuals with the power and skills to put it into practice, whether those are government officials and decision-makers, community groups and their representatives, or even nongovernmental organisations. Formal education, of course, has a key function here. But so does the informal education provided through the media. Furthermore, information and communications technologies (ICTs) have an important role to play in this process by reducing or eliminating the transactional (nonproduction) costs of communicating knowledge about science and technology.

At the same time, it is important that the practice of science communication reflects the fact that it takes place in social context. In other words, it is not just a question of conveying information, but also of engaging the potential users of that information. The need is to encourage dialogue and eventually to empower those to whom the information is being provided so that this information can be applied in a practical and useful way.

## SciDev.Net

It was with this in mind that the Science and Development Network (SciDev.Net) was launched in December 2001 as a source, through its Web site (www.scidev.net), of online news and analysis about the role of science and technology in meeting the needs of the developing world.

Much of the material we use is taken from the science journals *Nature* and *Science*, both of which provide us with free access for up to four articles each week, the selection being based on a decision about which articles—ranging from news items or editorials to full scientific papers—are directly relevant to the needs of developing countries. In addition, other news articles are contributed by staff writers and a growing team of correspondents, including science journalists in South Africa, India, Tanzania, Brazil, Colombia, and China. We also summarise and link to relevant news stories, feature items, and opinion articles from media outlets around the world.

An equally important part of the Web site are our dossiers. Dossiers are comprehensive, online guides to topical issues at the interface of science, technology, and society. Each dossier is compiled using advice from an international panel of experts and provides authoritative insight into a topic at the heart of international debate. So far, we have produced six dossiers on the following topics: intellectual property rights, climate change, indigenous knowledge, genetically modified crops, the ethics of biomedical research, and the brain drain. Those currently in the works focus on HIV/AIDS, research policy, genomics, and biodiversity.

## Target Audience

Our target audience is not easy to define, as it is made up of individuals from many different groups, from university researchers and teachers to government officials and aid agency staff. In broad terms, however, it can be taken to include all those with a professional or personal interest in the interactions between science, technology, and development. The number of registrants has risen steadily since the Web site was launched in December 2001 and now stands at more than 7,000. About half of our registered users come from the developing countries themselves. Of these, 43% come from Latin America—a reflection of various factors, including the size of its scientific community and its relatively high level of Internet connectivity. Sub-Saharan Africa comprise about 25%, and South Asia (mainly India) makes up 20%.

For those with an interest in science activities in a particular geographical region, we link together coverage into so-called regional gateways on the Web site. At present, there are four of these—covering Latin America, the Middle East, South and East Asia, and sub-Saharan Africa. Each gateway therefore has a strong regional identity, and in some cases this is reinforced by the translation of material—either in full text or as a summary—into local languages. The Latin American gateway, for example, carries three languages, and material can appear in one, two, or all three of these, with summaries in the others.

## Channels of Communication

Reflecting a commitment to the idea that scientific knowledge will only be effectively communicated if individuals are adequately trained to carry out this task, we are engaged not only in presenting information on our Web site, but also in helping to generate a capacity within developing countries to improve channels of communication for this knowledge.

One way in which we do this is through workshops on science communication. Some of these have a broad focus, seeking to help stimulate a national or regional debate on the importance of science communication, how it can be best carried out, and the challenges it faces. Such, for example, was the goal of two roundtables on the theme of “science communication and sustainable development” organised in August 2002 during the World Summit on Sustainable Development in Johannesburg, South Africa. Similarly, a meeting launching our activities in Latin America was held in Sao Paulo in May this year under the title “Science Communication and Development in Latin America.”

**Figure 1:**
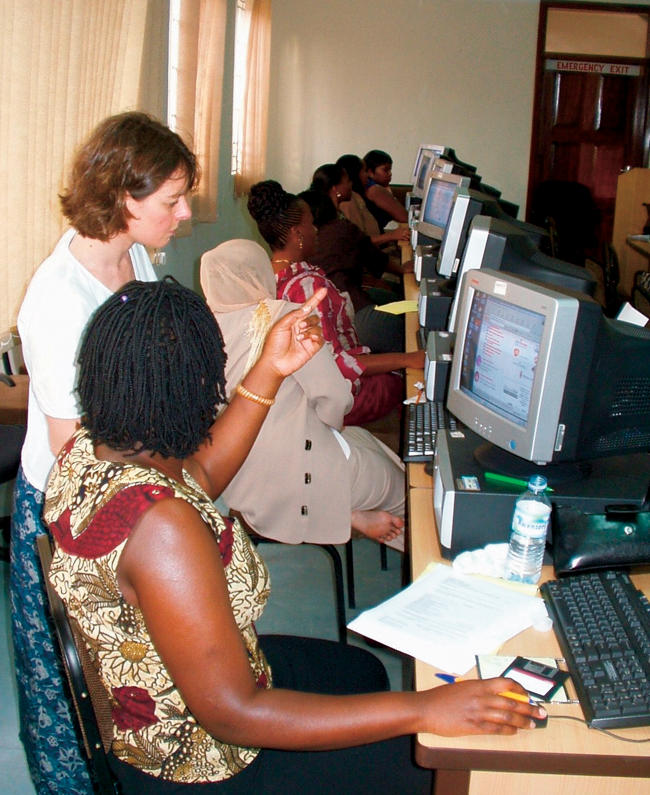
A Workshop on the Use of ICTs to Report on the Science of HIV/AIDS This workshop was held in Kampala, Uganda, in April 2003. (Photograph: SciDev.Net.)

Other events to encourage scientific communication have been more specific. In particular, we organised (jointly with the United Nations Educational, Scientific, and Cultural Organisation) a five-day workshop for African women communicators in Kampala, Uganda, in April 2003 on the use of ICTs to report on the science of HIV/AIDS (Figure 1). A similar event took place in Chennai, India, in November 2003, with participants from seven Asian nations.

## Partnerships

Building the communication channels that will allow science to be fully integrated into the development process at all levels—from governments down to communities—is a major challenge that involves many participants. SciDev.Net does not pretend to be more than one actor and is keen to establish partnerships with others in this field, convinced both of the synergies that can be gained through collaboration and of the contribution that ICTs can make to this (for example, by sharing information and links between Web sites).

One of the major challenges that we face is to build up our role as a platform for the voice of the developing world on the issues that we cover. We already do this to a certain degree through our correspondents, our regional networks of contributors, and the participation of scientists from developing countries who are on the advisory panels for our dossiers. But we are very aware that more effort is needed. Despite this, we already feel that we are beginning to make a mark. A staff member of the United States' National Academy of Sciences recently informed us: “We hosted a group of visitors from seven African scientific academies here in Washington last week, and they spoke highly of the SciDev project … you are becoming a truly global newsletter!”

## 

**Figure:**